# Mice with miR-146a deficiency develop severe gouty arthritis via dysregulation of TRAF 6, IRAK 1 and NALP3 inflammasome

**DOI:** 10.1186/s13075-018-1546-7

**Published:** 2018-03-15

**Authors:** Quan-Bo Zhang, Yu-Feng Qing, Cong-Cong Yin, Li Zhou, Xian-shuang Liu, Qing-Sheng Mi, Jing-Guo Zhou

**Affiliations:** 1 Department of Geriatrics, Affiliated Hospital of North Sichuan Medical College, 63 Wenhua Road, Nanchong, Sichuan 637000 People’s Republic of China; 2 Henry Ford Immunology Program, Department of Dermatology and Internal Medicine, Henry Ford Health System, One Ford Place, 1D-Rm. 31, Detroit, MI 48202-2689 USA; 3 Department of Rheumatology and Immunology, Affiliated Hospital, North Sichuan Medical College, 63 Wenhua Road, Nanchong, Sichuan 637000 People’s Republic of China; 4 Department of Internal Medicine, Affiliated Hospital of Qingdao University, Qingdao, Shandong People’s Republic of China; 5 Department of Neurology, Henry Ford Health System, Detroit, MI USA

**Keywords:** miR-146a, Gout, TRAF6, IRAK1, NALP3 inflammasome

## Abstract

**Background:**

MicroRNAs (miRNAs) serve as important regulators of inflammatory and immune responses and are implicated in several immune disorders including gouty arthritis. The expression of miR-146a is upregulated in the peripheral blood mononuclear cells of patients with inter-critical gout when compared to normouricemic and hyperuricemic controls and those patients with acute gout flares. However, the role of miR-146a in the development of gout remains unknown. Here, we used miR-146a knockout (KO) mice to test miR-146a function in a monosodium urate (MSU)-induced gouty arthritis model.

**Methods:**

The footpad or ankle joint of miR-146a KO and wild-type (WT) mice were injected with an MSU suspension to induce acute gouty arthritis. Bone marrow-derived macrophages (BMDMs) were stimulated with MSU and the gene expression of miR-146a; interleukin 1 beta (IL-1β); tumor necrosis factor-α (TNF-α); and the NACHT, LRR and PYD domains-containing protein 3 (NALP3) inflammasome was evaluated. TNF-α and IL-1β protein levels in BMDMs were assessed by fluorescence-activated cell sorting and western blot analyses. Gene and protein levels of TNF receptor-associated factor 6 (TRAF6) and IL-1 receptor-associated kinase (IRAK1), the targets of miR-146a, were also measured.

**Results:**

Significantly increased paw swelling and index and ankle joint swelling were observed in miR-146a KO mice compared to WT controls after MSU treatment. MiR-146a expression in BMDMs from WT mice was dramatically upregulated at 4 h following MSU stimulation. Additionally, the expression of IL-1β, TNF-α, and NALP3 was higher in BMDMs from miR-146a KO mice after exposure to MSU crystals compared to those from WT mice. Consistent with the observed gene expression, the IL-1β and TNF-α proteins were upregulated in miR-146a KO mice. Additionally quantitative RT-PCR and western blot demonstrated that TRAF6 and IRAK1 were dramatically upregulated in BMDMs from miR-146 KO mice compared to those from WT mice.

**Conclusions:**

Collectively, these observations suggest that miR-146a provides negative feedback regulation of gouty arthritis development and lack of miR-146a enhances gouty arthritis via upregulation of TRAK6, IRAK-1, and the NALP3 inflammasome function.

## Background

Gout is a prevalent disease manifesting most commonly as episodes of acute and extremely painful arthritis. Monosodium urate (MSU) crystals, a crystallized form of uric acid, deposit in joints and other tissues and induce the production of pro-inflammatory factors such as interleukin 1-beta (IL-1β), resulting in aseptic inflammation [1, 2].

MicroRNAs (miRNAs) constitute an abundant class of small, evolutionary conserved non-coding RNAs that function as post-transcriptional regulators [3]. Increasing evidence has demonstrated that miRNAs regulate gene expression by triggering translational inhibition and/or degradation of the targeted messenger in infectious and autoimmune diseases [4, 5]. One miRNA in particular, miR-146a, has been shown to act as a negative-feedback effecter in the inflammatory signaling pathway initiated by NF-κB [6], and it directly downregulates the production of pro-inflammatory cytokines by targeting TNF receptor-associated factor 6 (TRAF6) and IL-1 receptor-associated kinase (IRAK1), which are components of the cascade downstream of toll-like receptors and act as critical mediators of inflammation via impairment of NF-κB activity, to regulate innate immunity [6, 7]. Although the function and mechanism of miR-146a in many immune and rheumatic diseases has been investigated [6–9], little is known about its role in gouty arthritis. One report from Dalbeth et al. shows that miR-146a functions as a transcriptional break that is lost during acute inflammatory responses triggered by the presence of MSU crystals [10]. Unfortunately, the specific mechanisms of miR-146a regulates gouty arthritis were not explored in this study. Here, we sought to determinate the role of miR-146a and its mechanism of action in gouty arthritis using a miR-146a-deficient animal model.

## Methods

### Animals

MiR-146a knockout (KO) and B6 (wild-type (WT)) mice were housed at 24 ± 2 °C under a 12-h light/12-h dark cycle in a pathogen-free facility. Handing of mice and experimental procedures were in accordance with requirements of the Institutional Animal Care and Use Committee and this study was granted permission by the Ethics Committee of the Affiliated Hospital of North Sichuan Medical College.

### Preparing mouse bone marrow-derived macrophages (BMDMs) and MSU

Bone marrow cells were isolated from the femurs and tibias of the mice by flushing the medullary cavity with PBS containing 2% fetal calf serum (FCS). After one wash in the same solution, cells were seeded in petri dishes in PRIM-1640 medium supplemented with 20% FCS, 100 μg/mL streptomycin, 100 IU/mL penicillin, and 30 ng/mL macrophage colony-stimulating factor (BioSource International, Camarillo, CA, USA) at 37 °C for 7–9 days. Macrophages were then assessed by flow cytometry using a FACScan (Becton Dickinson Biosciences, San Jose, CA, USA) and staining with phycoerythrin (PE)-conjugated anti-F4/80 (eBioscience, San Diego, CA, USA) and fluorescein isothiocyanate (FITC)-conjugated anti-CD11b (eBioscience).

MSU crystals were prepared under pyrogen-free conditions. Briefly, 1 g uric acid (Sigma-Aldrich, St. Louis, MO, USA) was dissolved in 200 mL of boiling water containing 6 mL of 1 N NaOH. The pH value of the final solution was adjusted to 7.2 through the addition of HCl. The solution was cooled and stirred at room temperature and then stored overnight at 4 °C. The precipitate was filtered from the solution and dried under low heat. The crystals were weighed under sterile conditions and suspended in PBS at a concentration of 25 mg/mL [11].

### MSU-induced inflammation *in vivo*

Mice were injected intraperitoneally with 3 mg MSU crystals in 0.5 mL PBS. After 2.5 h and 5 h, the peritoneal cavities were washed with 2 mL PBS. The number of peritoneal exudate cells was counted using a hemocytometer. The cells were resuspended in PBS and subjected to staining and flow cytometric analysis.

### RNA extraction and quantitative reverse-transcribed PCR (qRT-PCR)

A total of 1 × 10^6^ BMDMs were treated with 0.25 mg/mL MSU crystals in a 24-well plate. At 0, 4, and 8 h after stimulation, total RNA was isolated from BMDMs using a combination of QIAzol lysis reagent and a miRNeasy Mini kit (Qiagen, Germantown, MD, USA) with some modifications. The RNA was reverse-transcribed (RT) using a TaqMan miRNA Reverse Transcription Kit (Applied Biosystems, Foster City, USA). Following pre-amplification, the gene expression was assessed on a 7900HT Fast Real-Time PCR System (Applied Biosystems), using the manufacturer’s recommended protocol. For analysis of gene expression, SYBR green gene expression assays were used for quantitative RT-PCR of IL-1β, TNF-α and NALP3. The primer sequences are shown in Table 1.

**Table 1:** The primers used for quantitative PCR

	Forward sequence (5′–3′)	Reverse sequence (5′–3′)
IL-1β	GGGCCTCAAAGGAAAGAATC	CTCTGCTTGTGAGGTGCTGA
TNF-α	ACAAAGGTGCCGCTAACCACATGT	ATGCTGCTGTTTCAGTCGAAGGCA
NALP3	CGTGGTTTCCTCCTTTTGTATT	CGACCTCCTCTCCTCTCTTCTT
ASC	TCACAGAAGTGGACGGAGTG	TGTCTTGGCTGGTGGTCTCT
Caspase-1	CGTGGAGAGAAACAAGGAGTG	AATGAAAAGTGAGCCCCTGAC
TRAF6	ATTTCATTGTCAACTGGGCA	TGAGTGTCCCATCTGCTTGA
IRAK1	GAGACCCTTGCTGGTCAGAG	GCTACACCCACCCACAGAGT
GAPDH	GGTGAAGGTCGGTGTGAACG	TGTAGACCATGTAGTTGAGGTCA

*NALP3* NACHT, LRR and PYD domains-containing protein 3, *ASC* apoptosis-associated speck-like protein containing a CARD, *TRAF6* TNF receptor associated factor 6, *IRAK1* interleukin-1 receptor-associated kinase, *GAPDH* glyceraldehyde-3-phosphate dehydrogenase

### ELISA

IL-1β levels in the BMDM culture supernatants were detected using a mouse IL-1β ELISA Ready-SET-Go! Kit (eBioscience), following the recommended protocol.

### Analyses of MSU-induced arthritis

A total of 1 mg MSU in 40 μL PBS or 0.5 mg MSU in 20 μL PBS was injected into the foot pads and synovial space of the right knee, respectively, of WT and miR-146a KO mice, while the same volume of PBS injected into the contralateral limb served as a control. The swelling index is expressed as the MSU-injected joint/PBS-injected joint ratio, and a ratio >1.15 indicated inflammation. Paw swelling and the size of the joint were measured with an electronic caliper by a researcher blinded to the intervention, at the indicated time points [12].

### Flow cytometric analysis

Single BMDM suspensions and peritoneal cells were incubated with mAb 2.4G2 for 30 min at 4 °C to block non-specific binding sites and then stained with mAbs anti-F4/80(eBioscience) and anti-CD11b(eBioscience) for 30 min at 4 °C. For intracellular staining, peritoneal cells were incubated with 100 μL IC fixation buffer (eBioscience) at 4 °C for 30 min in the dark. After incubation, cells were washed twice with 2 mL 1 × permeabilization buffer (eBioscience) and centrifuged and decanted. Next, intracellular staining was performed in 100 uL of 1 × permeabilization buffer using anti-TNF-a (eBioscience). After incubation for 30 min in the dark at room temperature, cells were washed twice with 1 × permeabilization buffer and resuspended in 150 μL 1 × PBS and filtered for flow cytometric analysis. Data were analyzed using a FACSAriaTMII (BD Biosciences, Franklin Lakes, NJ, USA) and Flowjo 7.6 DH software.

### Western blot analysis

The cells were disrupted in lysis buffer, and the concentrations of the extracted proteins were measured using a BCA Protein Assay Kit (Thermo Fisher Scientific, Rockford, IL, USA). The concentrations of the extracted proteins were measured using a BCA Protein Assay Kit (Thermo Fisher Scientific). The samples were separated on 10% SDS-PAGE and then electro-transferred at 90 V to an Immun-Blot polyvinylidene fluoride (PVDF) membrane for 2 h. Membranes were then blocked in I-BlockTM Protein-Based Blocking Reagent for 30 min at room temperature and then incubated with primary antibodies overnight at 4 °C. Blots were washed extensively in TBST and incubated with secondary antibodies for 2 h at room temperature. The signal was detected using an enhanced chemiluminescence method (ECL kit; Amersham Pharmacia Biotech, Piscataway, NJ, USA). All antibodies used were purchased from Santa Cruz Biotechnology (Santa Cruz Biotechnology, Santa Cruz, CA, USA).

### Data analysis

Data were analyzed using Prism 5 software (GraphPad Software, La Jolla, CA, USA). The results of gene expression were analyzed using the 2^-△△Ct^ method. Data from three to five individual experiments were pooled for analysis using the *t* test.

## Results

### MiR-146a expression is upregulated in WT BMDMs exposed to MSU crystals

**Fig. 1:**
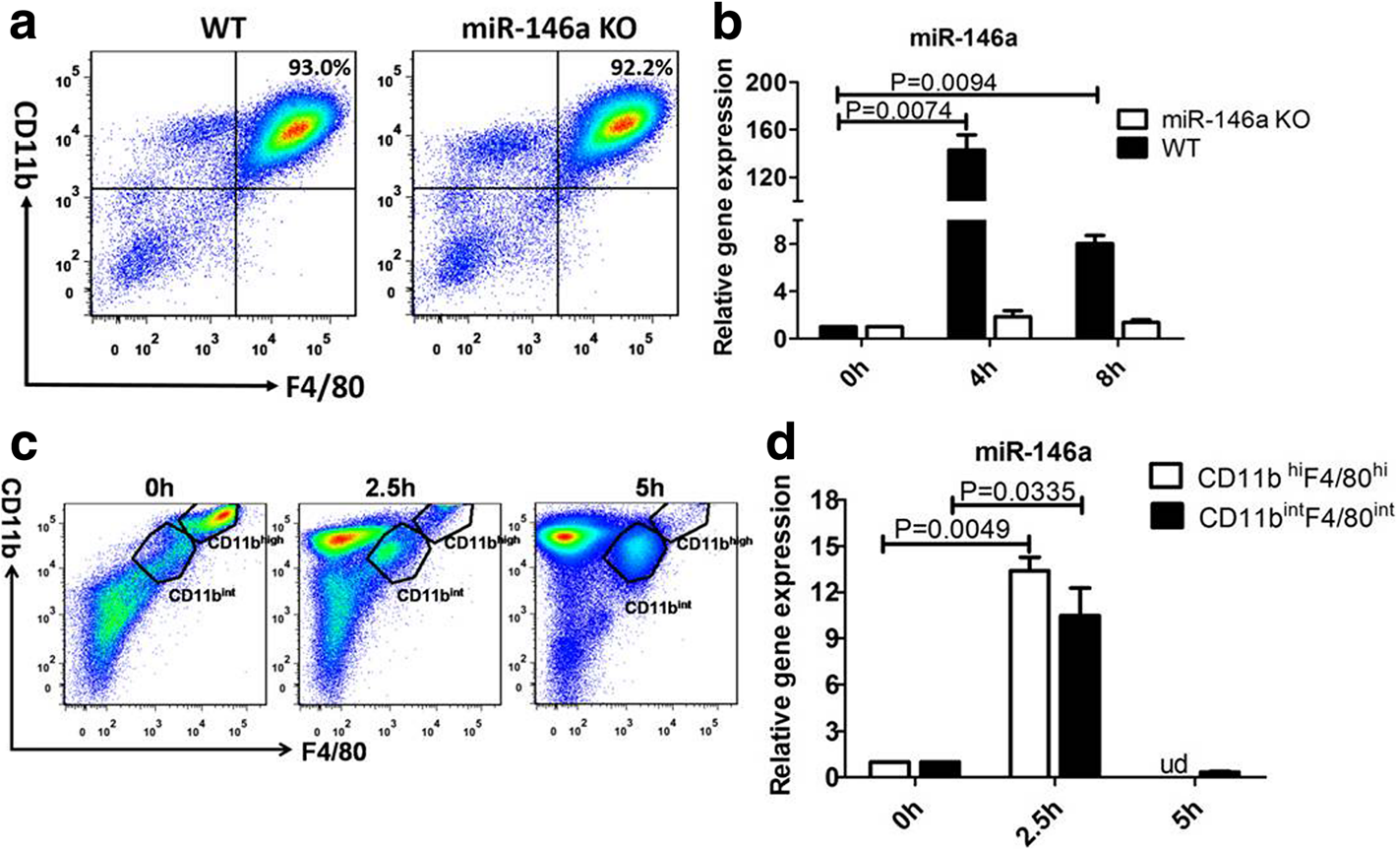
MiR-146a participates in monosodium urate (MSU)-induced inflammation **a**. The purity of bone-marrow-derived macrophages (BMDMs). Bone marrow cells of miR-146a knockout (KO) and wild-type (WT) B6 mice were cultured in RPMI-1640 medium containing 30 ng/mL macrophage colony-stimulating factor (M-CSF) for 7–9 days, and then co-stained with anti-F4/80 and anti-CD11b. The double-positive cells as the percentage of BMDMs among all cells is indicated (*n* = 3).**b** Relative expression of miR-146a following culture with 0.25 mg/mL MSU crystals for 0, 4, and 8 h. **c** The change in the two macrophage subsets. A total of 3 mg/0.5 mL MSU was injected into the peritoneal cavity; after 0, 2.5, and 5 h, peritoneal exudate cells were harvested and stained with anti-F4/80, and anti-CD11b, F4/80 + CD11b^high^, and F4/80 + CD11b^intmacrophage^ subsets were sorted: n = 3 for each group; groups were compared using the *t* test. **d** Relative expression of miR-146a in the sorted macrophages of the 3 mg/0.5 mL MSU-induced peritonitis model. ud, undetected

Bone marrow cells were induced with M-CSF 30 ng/mL for 7–9 days, and then co-stained with anti-F4/80 and anti-CD11b. The percentage of BMDMs, shown as double-positive stained cells, was more than 90% of all cells (Fig. 1a)**.** MiR-146a gene expression in WT BMDMs was upregulated 160-fold compared to unstimulated cells, following stimulation with MSU crystals at 4 h post-stimulation, and then decreased after 8 h (Fig. 1b). In the peritonitis model, miR-146a expression was upregulated in sorted CD11b^high^F4/80^high^ and CD11b^intermedium^F4/80^intermedium^ macrophage populations at 2.5 h after MSU injection, while the expression dropped to normal in CD11b^intermedium^F4/80^intermedium^ macrophage populations at 5 h post-injection. At this time point, the CD11b^high^F4/80^high^ subset and miR-146a expression was undetected (ud) (Fig. 1c, d). These results indicated that miR-146a was likely involved in the observed MSU-induced inflammation.

### MiR-146a KO mice suffer more severe arthritis than B6 mice

**Fig. 2:**
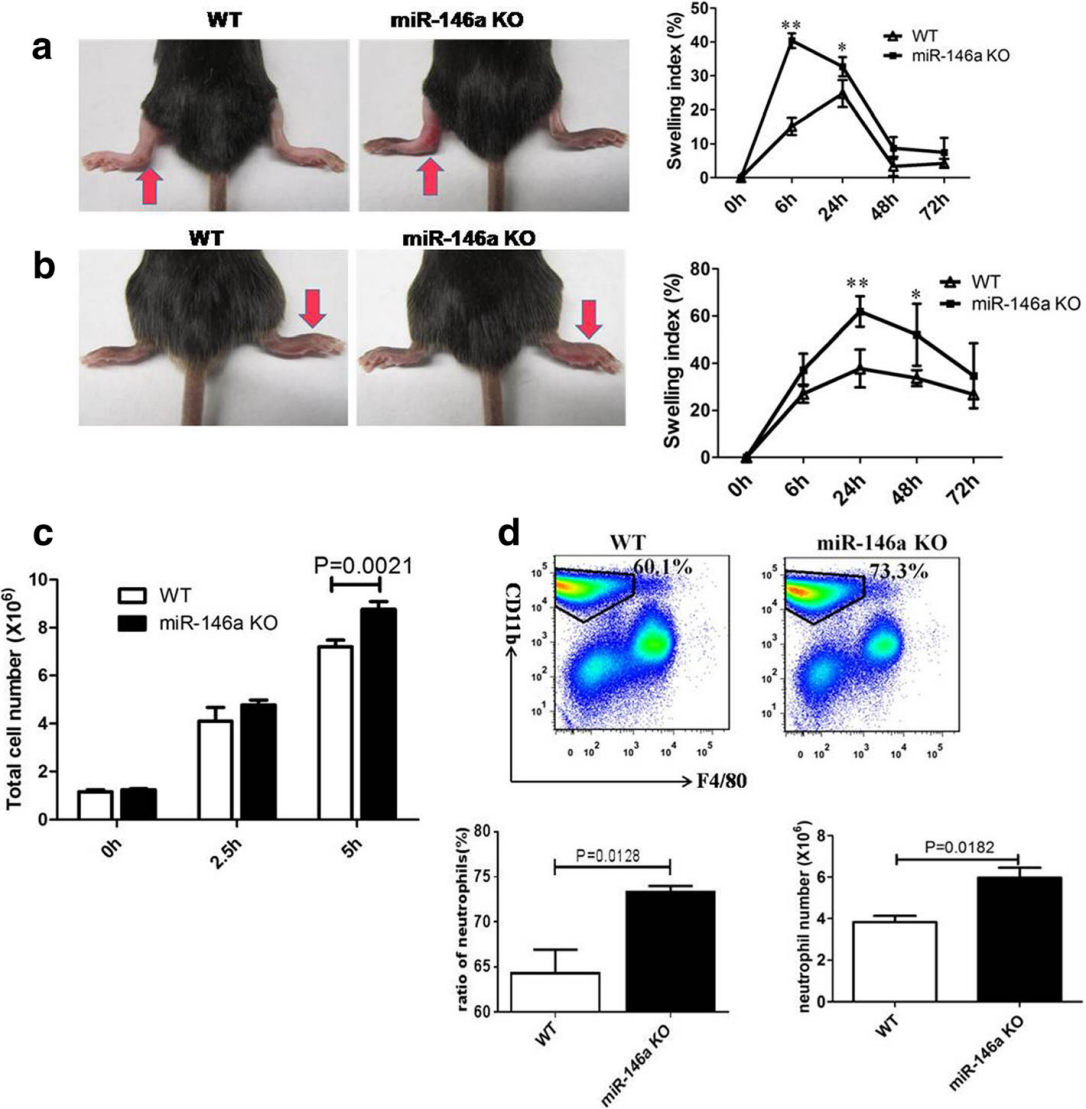
MiR-146a knockout (KO) mice suffer more severe arthritis than wild-type (WT) mice. **a** A total of 0.5 mg/20 μL monosodium urate (MSU) crystals were injected into the left rear ankle joints of miR-146a KO and WT B6 mice, while the same volume of PBS was injected into the right ankles. The swelling is expressed as the (left-right)/right ratio and a ratio >0.15 indicated inflammation. Data are expressed as mean ± SEM for five mice per group: **p < 0.01, *p < 0.05, *t* test. **b** A total of 1 mg/40 μL MSU suspension was injected into the right footpad of mice, and foot thickness was tested at 0, 6, 24, 48, and 72 h after MSU administration: *n* = 5 for each group, **p < 0.01, *p < 0.05, *t* test. **c** A total of 3 mg/0.5 mL MSU was injected into the peritoneal cavity, and after 2.5 h and 5 h the number of peritoneal exudate cells was counted using a hemocytometer; n = 3; groups were compared using the *t* test. **d** Peritoneal exudate cells were stained with anti-F4/80 and anti-CD11b; the F4/80^−^ CD11b ^+^ subset was gated as the neutrophil population; n = 3, groups were compared using the *t* test

MSU crystals were injected into the knee joint and footpad of the two strains of mice to mimic the etiologic origin of human gouty arthritis. A significantly increased paw swelling index was identified in miR-146a KO mice compared to WT controls at 6 h and 24 h after injection (Fig. 2a). Consistent with the observed increase in paw swelling, miR-146a KO mice also had more severe ankle joint swelling at 6 h, 24 h, and 48 h compared to WT mice (Fig. 2b). We also injected MSU into the peritoneal cavity of mice to construct a peritonitis model of gout and found total peritoneal cell numbers and neutrophil numbers were higher in miR-146a KO mice (Fig. 2c, d). These results implied that depletion of miR-146a enhanced neutrophil recruitment, boosting MSU-induced inflammation.

### Deficiency of miR-146a promotes MSU-induced pro-inflammatory cytokine expression in BMDMs and in peritoneal cells

**Fig. 3:**
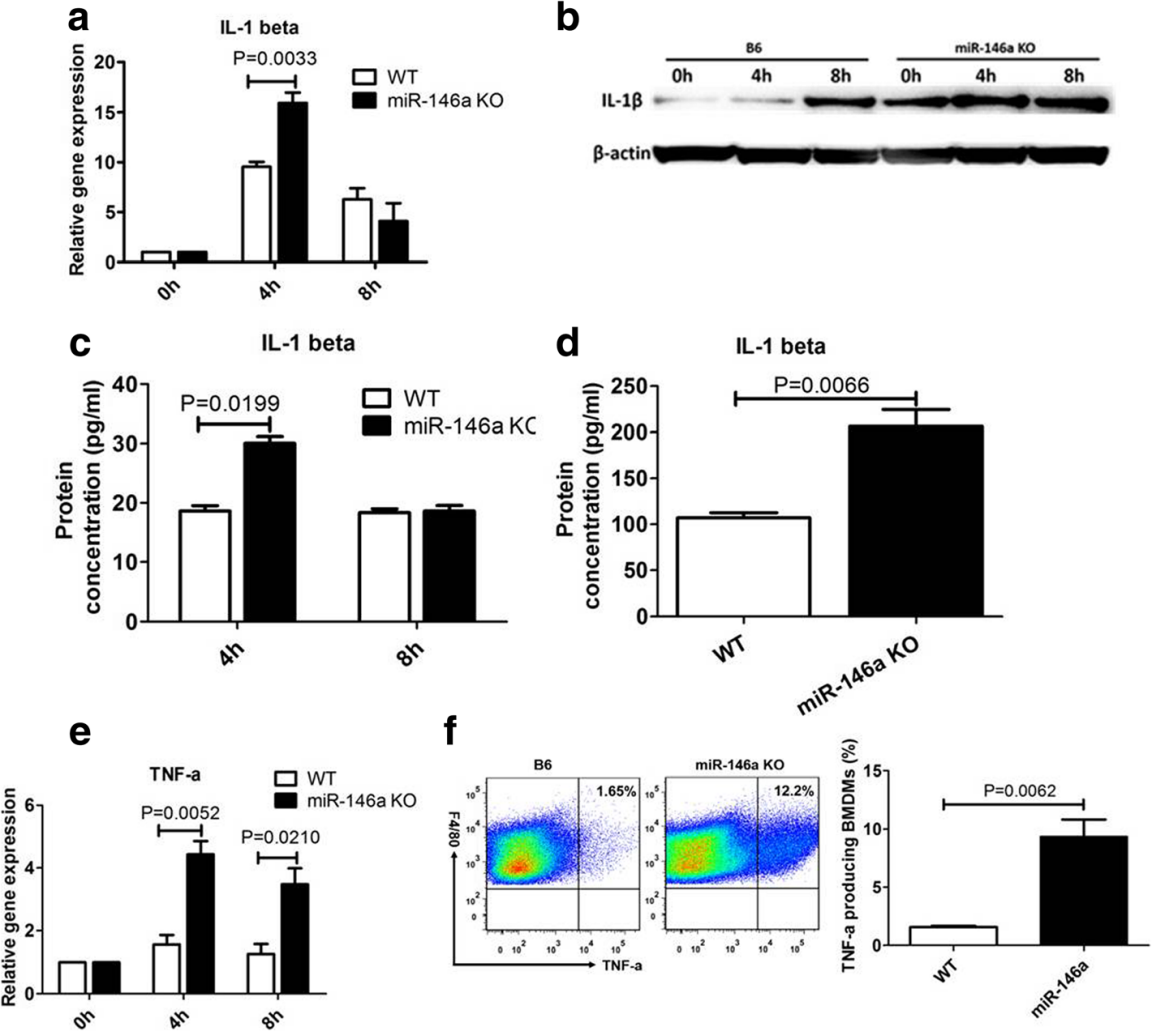
MiR-146a deficiency promotes synthesis and secretion of pro-inflammatory cytokines. Bone-marrow-derived macrophages (BMDMs) prepared from miR-146a knockout (KO) mice and wild-type (WT) B6 mice were incubated with endotoxin-free monosodium urate (MSU) crystals (0.25 mg/mL) for 0, 4, and 8 h. The IL-1β gene expression in BMDMs was detected via quantitative (q)PCR (**a**). IL-1β protein levels in BMDMs were measured by western blot (**b**). IL-1β protein levels were detected in BMDM culture medium (**c**) and in lavage fluid from the peritoneal cavity (**d**) using ELISA. TNF-α gene expression was measured by qRT-PCR in BMDMs (**e**). The ratio of BMDMs producing TNF-α was measured by fluroescence-activated cell sorting (FACS) (**f**). Values are the mean ± SEM of three to five individual experiments using cells from three different mice of each genotype; groups were compared using the *t* test

After validation (Fig. 1a), BMDMs from miR-146a KO and WT mice were exposed to MSU. The gene expression of IL-1β and TNF-α, typical pro-inflammatory cytokines present in gout, was enhanced in BMDMs after exposure to MSU crystals at 4 h and 8 h, and their expression was higher in BMDMs from miR-146a KO mice than WT mice (Fig. 3a, e). Consistent with these qPCR results, the western blot and ELISA analyses of IL-1β protein levels in BMDMs, culture supernatants, and peritoneal lavage fluids confirmed that BMDMs from miR-146a KO mice synthesized and secreted increased levels of mature IL-1β after exposure to MSU crystals (Fig. 3b-d). Additionally, western blot results showed that miR-146a deficiency markedly enhanced IL-1β secretion even in the absence of stimulation (0 h). FACS was used to analyze TNF-a protein production in BMDMs after treatment with MSU. As expected, the ratio of BMDMs producing TNF-a was higher in miR-146 KO mice than in the WT control (Fig. 3f).

### Mice with miR-146a deficiency develop severe gouty arthritis via dysregulation of TRAF6 and IRAK1

**Fig. 4:**
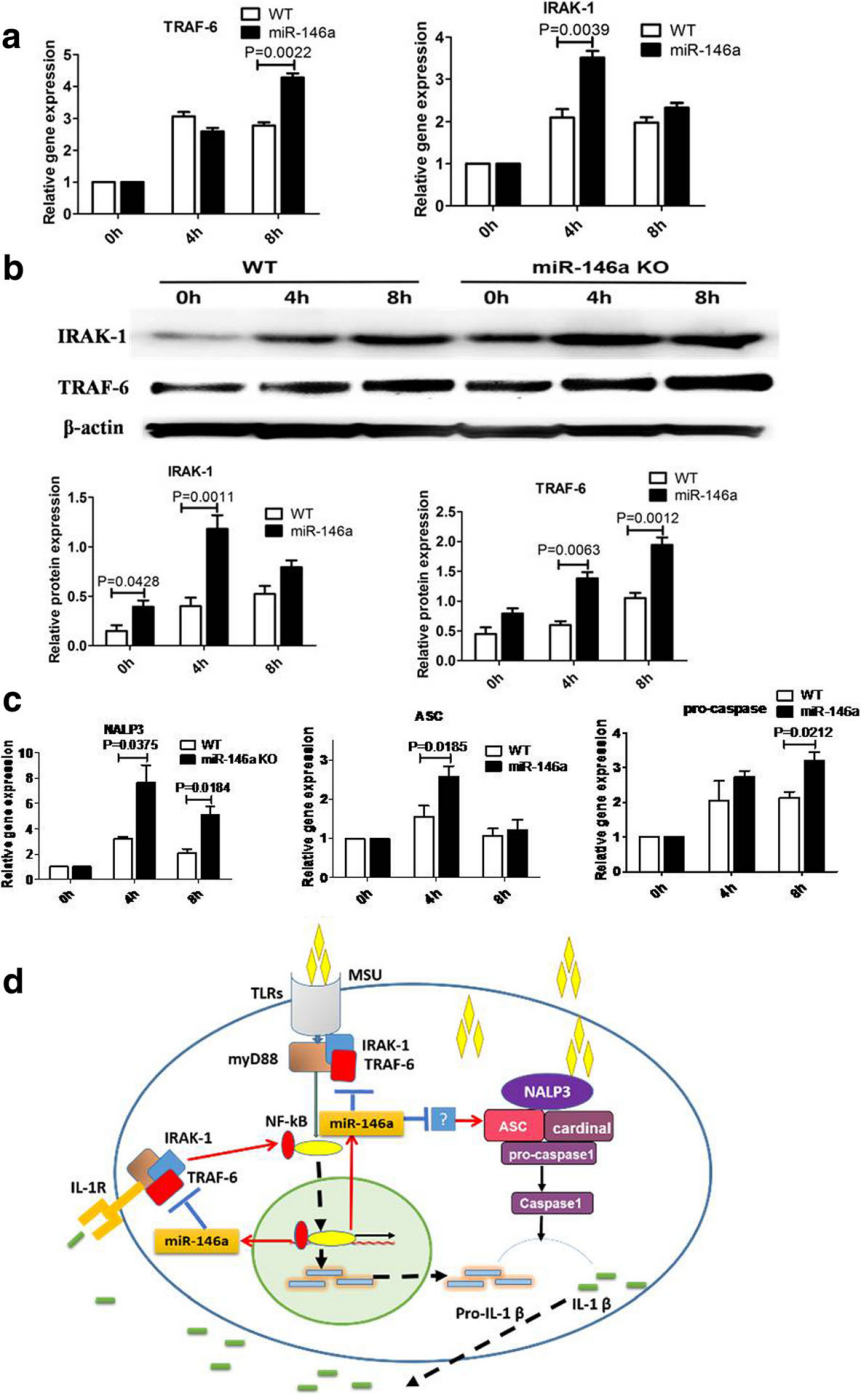
The potential mechanisms by which miR-146a influences gouty arthritis. TNF receptor associated factor 6 (TRAF6) and interleukin-1 receptor-associated kinase (IRAK1) gene and protein levels were measured by quantitative (q)RT-PCR (**a**) and western blot (**b**), respectively, in bone-marrow-derived macrophages (BMDMs) from miR-146a knockout (KO) and wild-type (WT) B6 mice exposed to 250 μg/mL monosodium urate (MSU) for 0, 4, and 8 h. **c** qPCR was used to measure NACHT, LRR and PYD domains-containing protein 3 (NALP3), apoptosis-associated speck-like protein containing CARD (ASC), and pro-caspase 1 gene levels in BMDMs exposed to 250 μg/mL MSU for the different time periods indicated. **d** Schematic shows how miR-146a might decrease IL-1β production by inhibition of TRAF6 and IRAK1 function. Values are the mean ± SEM of three individual experiments using cells from three different mice of each genotype; groups were compared using the *t* test. TLRs, toll-like receptors.

To explore the mechanism of enhanced gouty arthritis occurring in miR-146a KO mice, the gene and protein expression of TRAF6 and IRAK1, the direct targets of miR-146a, were detected using qRT-PCR and western blot. As shown in Fig. 4, the proteins level of TRAF6 and IRAK1 were dramatically upregulated in the BMDMs of miR-146 KO mice (Fig. 4b), a tendency that was consistent with the observed change in gene expression assessed via qRT-PCR (Fig. 4a).

### Increased expression of NALP3 inflammasome components in MSU-induced BMDMs of miR-146 KO mice

The NALP3 pathway also plays a vital role in the pathophysiology of acute gout. Pro-IL-1β is cleaved to its mature form by caspase-1, and the NALP3 inflammasome is essential for caspase-1 activation. Even though the NALP3 inflammasome is not a direct target of miR-146a, we assessed the gene expression of components of the NALP3 inflammasome, NALP3, apoptosis-associated speck-like protein containing a CARD (ASC), and caspase-1, in MSU-induced BMDMs. Surprisingly, we found that the gene expression of NALP3, ASC and caspase-1 were dramatically upregulated in BMDMs of miR-146a KO mice compared with WT mice exposed MSU crystals (Fig. 4c).

## Discussion

This study has shown that miR-146a KO mice suffer more severe gouty arthritis than WT mice and has indicated that miR-146a-deficient mice lose the repression of TRAF6 and IRAK1, leading to enhanced production and secretion of pro-IL-1β. To our knowledge, the current study is the first to identify the importance of miR-146a in gouty arthritis using a KO mouse model and to explore the potential mechanism underlying this relation.

Previous studies have shown that miR-146a is upregulated in lipopolysaccharide (LPS) or MSU stimulated THP-1, a common human monocyte cell line used to study monocyte/macrophage differentiation and function [7, 10]. In our study, miR-146a expression was dramatically upregulated in BMDMs after exposure to MSU crystals for 4 h, which is in agreement with the study by Dalbeth et al. [10]; interestingly, we were surprised to observe that miR-146a expression was sharply downregulated at 8 h post-stimulation. Furthermore, miR-146a expression paralleled the gene expression of IL-1β, suggesting that miR-146a plays a pivotal role in MSU-induced inflammation and that its role might be closely connected with that of IL-1β. A previous study has shown that peritoneal monocyte miR-146a expression is significantly reduced at 2 h and 8 h following injection of MSU [10]. We stained MSU-induced peritoneal cells with anti-F4/80 and anti-CD11b antibodies and obtained two macrophage subsets, CD11b^high^F4/80^high^ and CD11b^intermedium^F4/80^intermedium^. Additionally, miR-146a expression was significantly upregulated at 2.5 h following injection, with normalization of expression at 5 h. These findings were in contrast with the study of Dalbeth et al. [10], perhaps reflecting that sorted macrophages can more accurately represent the inflammatory response in MSU-induced inflammation.

To further confirm the participation of miR-146a in MSU-induced inflammation. MSU crystals were injected into the ankle joints and footpads of WT and miR-146a KO mice to mimic acute gouty arthritis in humans; the data indicated that miR-146a KO mice suffered more severe arthritis than WT mice in two types of gouty models, signifying miR-146a acts as a negative regulator during the process of gouty arthritis.

MiR-146a can directly downregulate the production of pr-inflammatory cytokines, which play critical roles in the inflammatory responses triggered by MSU crystals, by acting as a negative-feedback effecter of the inflammatory signaling pathway initiated by NF-κB [4]. Our results showed that miR-146a deficiency led to enhanced IL-β and TNF-α expression in MSU-primed BMDMs. Meanwhile, we also found that IL-1β protein levels were much higher in miR-146a-deficient BMDMs than in WT cells, even without stimulation by MSU, implying that loss of miR-146a may lead to an autoimmune phenotype and exaggerate the inflammatory response in mice [9, 11]. Previous studies have suggested that IL-1β acts as the central regulatory cytokine in acute gouty arthritis to recruit neutrophils into the synovium and joint [12]. As we know now, the production and activity of IL-1β are tightly regulated via a multi-step process. The precursor of IL-1β (pro-IL-1β) is synthesized based on the activation of the toll-like receptor (TLR) pathway, and pro-IL-1β is then cleaved to a mature form by the NALP3 inflammasome [1, 13]. It is has been proposed that miR-146a targets IRAK1 and TRAF6, two central adaptor kinases in the downstream signaling cascade of TLR, mediating the NF-κB pathway through a negative feedback regulation loop, leading to an obvious reduction in the pro-inflammatory cytokines IL-1β, TNF-α, and IL-6 in mycobacteria-infected macrophages [14–16]. Consistent with previous reports [7, 14, 15], deficiency of miR-146a in BMDMs exposed to MSU led to a significant increase in gene and protein expression of both TRAF6 and IRAK1 compared with the WT control. Synthesized the results of IL-1β and TNF-α changing tendency, it is indicated that knock out miR-146a discharged the repression of TRAK6 and IRAK1 function to accelerate the production pro-inflammatory cytokines and to exacerbate the MSU-induced gouty arthritis (Fig. 3c).

MSU crystals can trigger the activation of the NALP3 inflammasome, culminating in the production of IL-1β. We were surprised to find increased mRNA levels of NALP3 inflammasome components in macrophages of miR-146a KO mice, as there are no reports indicating that miR-146a directly targets components of NALP3 inflammasome, and bioinformatics analysis has indicated that NAPL3 does not contain a binding site for miR-146a. Our results suggest the possibility that miR-146a indirectly targets the NALP3 inflammasome to improve the activation of IL-1β; alternatively, some unknown regulatory mechanisms may exist to promote this effect.

## Conclusions

MiR-146a has been shown to serve as an important regulator of inflammatory and immune responses and is implicated in several immune disorders including gouty arthritis. However, the mechanism of miR-146a regulating gout development remains unknown. Here, we used miR-146a knockout (KO) mice to test miR-146a function in MSU-induced gouty arthritis and our data suggest that miR-146a provides feedback regulation of gout arthritis development and lack of miR-146a enhances gouty arthritis through upregulating TRAK6/IRAK1 and NALP3 inflammasome function (Fig. 4d). The results indicate that targeting miR-146a in macrophages may be an effective therapeutic strategy in the treatment of gout.

## Abbreviations

ASC: Apoptosis-associated speck-like protein containing a CARD
BMDMs: Bone-marrow-derived macrophages
ELISA: Enzyme-linked immunosorbent assay
FACS: Fluoresence-activated cell sorting
FCS: Fetal calf serum
IL-1β: Interleukin 1 beta
IRAK1: Interleukin-1 receptor-associated kinase
KO: Knockout
LPS: Lipopolysaccharide
mABs: Monoclonal antibodies
M-CSF: Macrophage colony-stimulating factor
miRNAs: MicroRNAs
mRNA: Messenger RNA
MSU: Monosodium urate
NALP3: the NACHT LRR and PYD domains-containing protein 3
NF-κB: Nuclear transcription factor-κB
PBS: Phosphate-buffered saline
qPCR: Quantitative polymerase chain reaction
TLR: Toll-like receptor
TNF-α: Tumor necrosis factor-α
TRAF6: TNF receptor associated factor 6
ud: Undetected
WT: Wild-type
